# Deep Hybrid Convolutional Neural Network for Segmentation of Melanoma Skin Lesion

**DOI:** 10.1155/2021/9409508

**Published:** 2021-11-08

**Authors:** Cheng-Hong Yang, Jai-Hong Ren, Hsiu-Chen Huang, Li-Yeh Chuang, Po-Yin Chang

**Affiliations:** ^1^Department of Electronic Engineering, National Kaohsiung University of Science and Technology, Kaohsiung 80778, Taiwan; ^2^Program in Biomedical Engineering, Kaohsiung Medical University, Kaohsiung 80708, Taiwan; ^3^School of Dentistry, Kaohsiung Medical University, Kaohsiung 80708, Taiwan; ^4^Department of Community Health Physical Medicine and Rehabilitation Physician, Chia-Yi Christian Hospital, Chia-Yi City 60002, Taiwan; ^5^Department of Chemical Engineering and Institute of Biotechnology and Chemical Engineering, I-Shou University, Kaohsiung 84001, Taiwan

## Abstract

Melanoma is a type of skin cancer that often leads to poor prognostic responses and survival rates. Melanoma usually develops in the limbs, including in fingers, palms, and the margins of the nails. When melanoma is detected early, surgical treatment may achieve a higher cure rate. The early diagnosis of melanoma depends on the manual segmentation of suspected lesions. However, manual segmentation can lead to problems, including misclassification and low efficiency. Therefore, it is essential to devise a method for automatic image segmentation that overcomes the aforementioned issues. In this study, an improved algorithm is proposed, termed EfficientUNet++, which is developed from the U-Net model. In EfficientUNet++, the pretrained EfficientNet model is added to the UNet++ model to accelerate segmentation process, leading to more reliable and precise results in skin cancer image segmentation. Two skin lesion datasets were used to compare the performance of the proposed EfficientUNet++ algorithm with other common models. In the PH2 dataset, EfficientUNet++ achieved a better Dice coefficient (93% vs. 76%–91%), Intersection over Union (IoU, 96% vs. 74%–95%), and loss value (30% vs. 44%–32%) compared with other models. In the International Skin Imaging Collaboration dataset, EfficientUNet++ obtained a similar Dice coefficient (96% vs. 94%–96%) but a better IoU (94% vs. 89%–93%) and loss value (11% vs. 13%–11%) than other models. In conclusion, the EfficientUNet++ model efficiently detects skin lesions by improving composite coefficients and structurally expanding the size of the convolution network. Moreover, the use of residual units deepens the network to further improve performance.

## 1. Introduction

Melanoma is a type of skin cancer with high spread characteristics and a mortality rate of approximately 75% [1]. Machine learning algorithms have been widely used in cancer research [2–5]. Moreover, medical image segmentation and computer-aided vision techniques have recently been used to improve the diagnosis of cancer lesions [6–8]. Image segmentation plays a vital role in many medical imaging applications, and it can conveniently and automatically describe the contours of anatomical structures and other regions of interest.

Convolutional neural networks (CNNs) are the most commonly used algorithms in medical imaging [9–11] and are used for many tasks, including image classification [10, 12, 13], superresolution [14–16], object detection [17–19], and semantic segmentation [20–22]. However, image segmentation is a considerable barrier for precise computer-aided diagnoses. Image segmentation is different from image classification or object recognition in that it is not necessary to know beforehand what visual concepts or objects are being analyzed [23]. Deep learning can be used to automatically extract features from images in different categories; this may improve the feature detection time and efficiency of traditional computer-aided detection by 10%.

Zhang et al. improved the accuracy of neural network image segmentation using a SENet module with a U-Net encoder [24]. The SENet module demonstrably improved feature extraction and reduced running time. Xiuqin et al. added a residual module based on the U-Net network, which increased model performance in two aspects, namely, by (1) improving the performance of network training and reducing the gradient drop problem and (2) using the jump connection residual module to nondegradation of information and thereby allow deeper network structures to be designed, which improved semantic segmentation performance [25]. Wei et al. overcame the problem of limited available dermoscopic image datasets using a pretrained network and transferring the model parameters of the DenseNet161 model trained on an ImageNet dataset for natural image classification to Segmentor's downsampling path architecture [26]. Goyal et al. proposed two ensemble methods called Ensemble-ADD and Ensemble-Comparison to improve segmentation performance [27]. First, if no DeeplabV3+ prediction is available, the ensemble methods pick up the prediction of Mask R–CNN and vice versa. Then, Ensemble-ADD combines the results of both Mask R–CNN and DeeplabV3+ to produce the final segmentation mask. Ensemble-Comparison-Large picks the larger segmented area by comparing the number of pixels in the outputs of both methods. By contrast, Ensemble-Comparison-Small picks the smaller area from the output.

The research field of automatic prostate segmentation in 3D MR images presents challenges. The lack of exact edges between the prostate and other anatomical structures makes the challenge on accurate boundary extraction. The segmentation is further complicated by the complex background texture and the significant variation in the prostate's size, shape, and intensity distribution. Zhu et al. [28] proposed BOWDA-Net, a boundary-weighted domain self-tuning neural network, to solve the problem for small medical imaging datasets [28]. Zhu et al. [29] suggested a novel 3D network with a self-supervised function entitled Selective Information Transfer Network (SIP-Net). They assessed the suggested model on the MICCAI Prostate MR Image Segmentation 2012 Grant Challenge dataset, TCIA Pancreas CT-82, and MICCAI Liver Tumor Segmentation (LiTS) 2017 Challenge datasets. The empirical results of these datasets show that the proposed model obtains more reliable segmentation results and outperformed state-of-the-art methods recently [29]. In the work by Liu et al. [30], a new learning approach and multisite-guided knowledge transfer was used to overcome the difficulty of acquiring shared knowledge from multiple datasets. This method revealed to enhance the kernel to extract more common representations from multisite data. Extensive experiments on three heterogeneous prostate MRI datasets show that our MS network consistently improves the performance on all datasets and outperforms state-of-the-art multisite learning methods.

## 2. Methods

**Figure 1:**
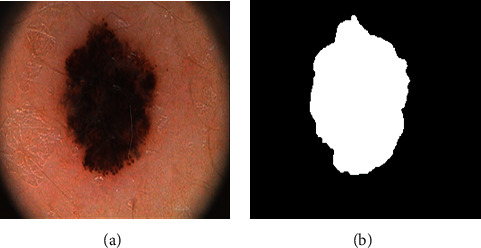
Skin lesion samples (a) and corresponding masks (b).

Melanoma is a type of skin cancer with high spread characteristics and a mortality rate of approximately 75% [1]. Machine learning algorithms have been widely used in cancer research [2–5]. Moreover, medical image segmentation and computer-aided vision techniques have recently been used to improve the diagnosis of cancer lesions [6–8]. Image segmentation plays a vital role in many medical imaging applications, and it can conveniently and automatically describe the contours of anatomical structures and other regions of interest.  Figure 1 shows images of a skin lesion and manually segmentation mask.

### 2.1. U-Net

**Figure 2:**
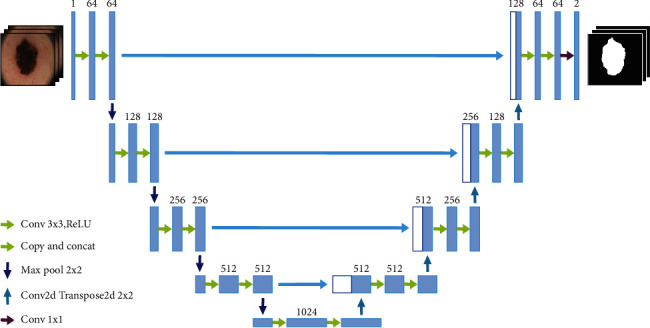
U-Net structure diagram.

U-Net for segmentation is illustrated in Figure 2 [31] The first half of the U-Net network performs feature selection, and the second half performs upsampling. This network structure is also called a transcoder. In the first half of the U-Net encoding path, convolution and max pooling are repeated. After every two 3 × 3 convolutional layers on the path, a 2 × 2 max pool layer is added. After layer convolution, the rectified linear unit (ReLU) activation function is used to downsample the original image. Each downsampling adds a new number of channels, and each layer step in the second half of the decoding path involves continuously performing transposed convolution and convolution operations on the feature map. In the upsampling half of the decoding path, an ReLU activation function and two 3 × 3 convolutional layers are added after each layer of transposed convolution.

Each upsampling adds a feature map of the corresponding encoding path, thereby reducing the number of feature channels by half. For transposed convolution, the general convolution is reversed, and the feature map obtained using convolution is restored to the pixel space through transposed convolution. The largest corresponding feature map can intuitively understand which features are selected through a convolution operation. The last layer of the network is a 1 × 1 convolutional layer, which can convert the 64-channel feature vector into the required number of classification results. U-Net can perform convolution operations on images of arbitrary shapes. The U-Net encoding path is upsampled four times and downsampled 16 times. Because of four upsampling operations, detailed information, such as that on the edge restoration of the segmentation map, can be obtained, enabling the decoder to determine target details, achieve point-level positioning, and devise decoding paths. The operations also correspond to four upsamplings.

In U-Net, low-level and high-level information is combined. After multiple downsamplings of the low-level information, the low-resolution information can provide contextual semantic information of the segmentation target in the entire image, reflecting the characteristics of the target and its relationship with the environment. This feature determines the object category and uses skip connections between the encoder and decoder. The skip connection operation applied to high-level information involves directly passing information from the encoder to high-resolution information decoded at the same bandwidth, which provides more detailed features for segmentation. When low-level information is combined with high-level information, directly supervising semantic features and loss backpropagation is not necessary. The recovered feature map incorporates more low-level information features and features at different scales. Unlike other networks, U-Net uses feature fusion to perform stitching and stitches features together in channel dimensions to form thicker features.

The U-Net model for medical image segmentation has the following advantages: (1) The boundaries of medical images are blurred, and gradients are complex. Moreover, more high-resolution information is required. High-resolution information is mainly used for accurate segmentation. (2) The internal structure of the human body is relatively fixed. The distribution of segmentation targets in human images is regular, and the semantics are simple and clear. Low-resolution information is suitable for target recognition. Such information is combined with high-resolution information in U-Net.

### 2.2. SegNet

**Figure 3:**
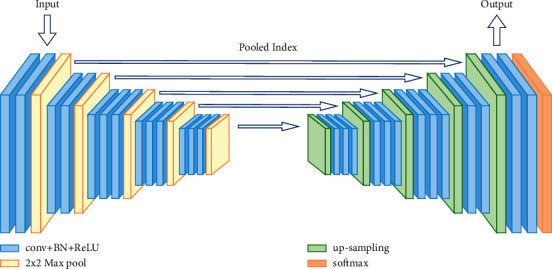
SegNet structure diagram.

The SegNet [32] architecture consists of a sequence of nonlinear processing layers (encoders) and a corresponding set of decoders followed by a pixelwise classifier. Typically, each encoder consists of one or more convolutional layers with batch normalization and ReLU nonlinearity, followed by nonoverlapping max pooling and subsampling. The sparse encoding due to the pooling process is upsampled in the decoder using the max pooling indices in the encoding sequence (Figure 3). One key feature of SegNet+ is the use of max pooling indices in the decoders to perform upsampling of low-resolution feature maps. This has the notable advantages of retaining high-frequency details in the segmented images and of reducing the total number of trainable parameters in the decoders. The entire architecture can be trained end-to-end using stochastic gradient descent. The raw SegNet predictions tend to be smooth even without conditional random field-based postprocessing.

### 2.3. UNet++ and Improved UNet++

**Figure 4:**
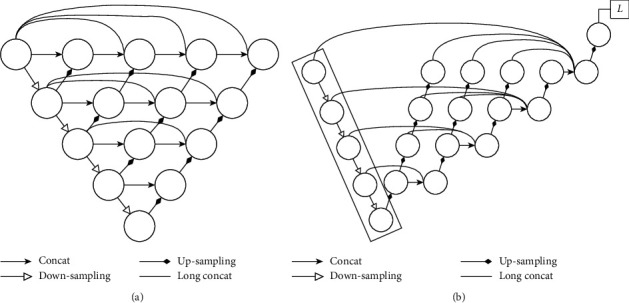
Structure diagram of (a) UNet++ module and (b) improved UNet++ module. *L* denotes loss function.

As depicted in Figure 4, UNet++ [33] has some improvements over U-Net [31]; such improvements mainly relate to the skip connection part of the U-Net structure. Compared with the original U-Net network, UNet++ connects layers 1 to 4 of U-Net together. The advantages of this structure are as follows: (1) Regardless of whether the depth feature is effective, it will still be used so that the network can learn the importance of features of different depths. (2) As the feature extractor is shared, the entire U-Net need not be trained: only one encoder is trained, and the features of different levels are restored by different decoder paths. In the UNet++ architecture, the encoder can be flexibly replaced with various backbones. The main improvement of UNet++ is in filling up the original, hollow U-Net: the advantage is in grasping different levels of features and using feature overlay to integrate different levels of features so that when semantically similar feature maps are received, the semantic gap between the encoder and decoder feature stubs is reduced, making optimization easier. Sun et al. [34] proposed a new architecture called UNet+, which is formed by removing the original skip connection in U-Net and connecting every two adjacent nodes in the set. Based on the new connection scheme, UNet + connects disjoint decoders, thus enabling gradient backpropagation from deep decoders to shallow decoders. UNet + further relaxes the unnecessary restrictive behavior of skip connections by proposing an ensemble of all feature mappings computed in the shallower stream [34]. Therefore, removed dense connections is a neural network adjustment technique, which enables gradient backpropagation from deep decoders to shallow decoders. In the structure of improved UNet++, we removed dense connections from the original UNet++ to reduce the amount of calculation and change the way of skip connections.

### 2.4. High-Resolution Network

**Figure 5:**
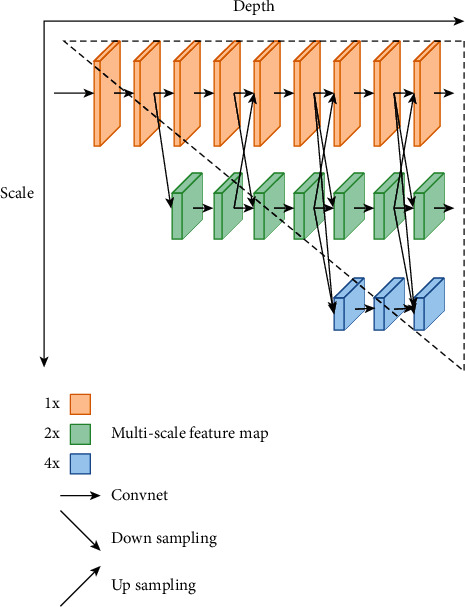
The neural network architecture of HRNeT.

U-Net [31], SegNet [32], and other methods have been widely applied in many studies; their features are using convolutional operations to compute low-resolution representations and then gradually recover high-resolution representations. However, in order to learn high representations and reduce spatial accuracy loss, HRNet employs a strategy of maintaining high resolution throughout the feature extraction process [35]. The architecture consists of a multiresolution convolutional parallel module, an interactive fusion module, and a representation head module. Sun et al. [34, 35] augmented HRNet with a direct segmentation head. He aggregated the output representations at four different resolutions and then used 1 × 1 convolution to fuse these representations. The HRNeT architecture is shown in Figure 5. The output representations are fed into the classifier. Ke Sun evaluated his method on three datasets, namely, Cityscapes, PASCAL-Context, and LIP, and achieved state-of-the-art performance. Therefore, we introduced HRNeT to image segmentation applications for skin lesions in order to benchmark the performance of the model. Figure 5 indicates that the neural network architecture of HRNeT is composed of parallel high- and low-resolution subnetworks, and the multiscale feature fusion that 1x, 2x, and 4x is achieved by repeatedly exchanging information between multiresolution subnetworks [35]. The horizontal and vertical directions in the figure correspond to the depth of the network and the scale of the feature map, respectively.

### 2.5. Backbone

Transfer learning is a machine learning technique where for which the knowledge gained in training one problem is used in the training of another task or domain [36]. In deep learning, the first few layers are trained to define the features of the task. In transfer learning, the last few layers of the trained network can be removed, and new layers are used to retrain the target task. In the transfer learning approach, using the network knowledge previously trained with a large amount of visual data for a new task is very beneficial in saving time and achieving high accuracy compared with training the model from scratch.

This study proposed EfficientUNet++ that replaced the encoder in UNet++ with two backbone architectures: pretrained Xception [37] and EfficientNet [38] models. Although for both architectures, networks of varying depths exist, we choose the shallower depths to prevent overfitting to our limited training data. These models have learned to extract useful and powerful features from images and use them as starting points for learning new tasks. They use pretraining as a benchmark for improving existing models while adding the advantages of pretrained models to make learning efficiency faster and more stable and to accurately split skin lesion images.

#### 2.5.1. Xception

**Figure 6:**
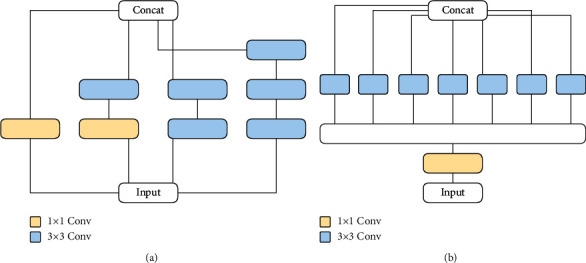
The network architecture for (a) Inception module and (b) Xception module.

Xception as depicted in Figure 6 and the Inception model [39] can determine the correlation between functional channels and the spatial correlation between separate channels of the function using convolution operations. Xception [37] achieves higher recognition accuracy than the Inception model. The structure consists of 36 convolutional layers for the characteristic extraction of the network. The 36 convolutional layers are divided into 14 modules, and the above structure is redesigned as an inception model block. In the case of redesigning the ResNet architecture, it is possible to increase the number of layers of the model while reducing the number of parameters. This not only reduces storage space but also enhances the expressive power of the model. Each 3 × 3 convolution acts on a feature map containing one channel only; this is the basic module of Xception. Adding a residual connection mechanism similar to ResNet to Xception significantly accelerates the convergence process and achieves significantly higher accuracy.

#### 2.5.2. EfficientNet

**Figure 7:**
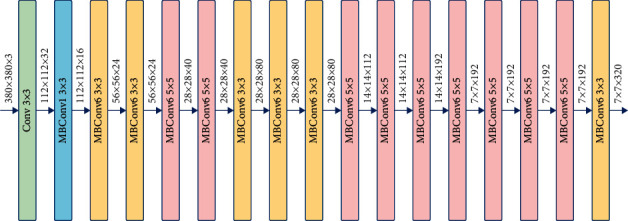
Architecture of EfficientNet B0.

As shown in Figure 7, EfficientNet [38] uses MBConv in MobileNetV2 [40] as the backbone network of the model. It also uses the extrusion and excitation methods in SENet to optimize the network. It can be expanded from B0 to B7 using the compound expansion method, together with adjustment of scaling parameters to increase the size of the network in order to improve accuracy.

### 2.6. EfficientNet++ Training

EfficientNet++ was tested on two skin lesion segmentation datasets with a small number of dermatoscopic images in the training set compared with the number of network parameters. Therefore, to improve the performance of EfficientNet++ and overcome the possible overload problem due to the insufficient training set, the following three strategies were used for the segmentation of dermatoscopic images.

#### 2.6.1. Preprocessing

A standard preprocessing step was performed on the images before delivering them to the network. Initially, an algorithm for grayscale world color constancy was applied to normalize the image's color, as suggested in [41]. This preprocessing step deals with varying lighting conditions in the images and is widely used for skin lesion analysis [42–44]. Subsequently, as a common preprocessing step for transfer learning, the mean intensity RGB values were subtracted from the ImageNet dataset [45]. Various enhancement techniques were used to obtain more robust models, including random scaling, random rotation, vertical and horizontal flipping, random luminance and contrast shifts, random adaptive histogram equalization, random cropping, and random manipulation of HSV (Hue, Saturation and Value) color channels. The random cropping strategy is applied to both the training and validation set images to calculate the scores.

#### 2.6.2. Dropout and Batch Normalization

The motivation of using the batch normalization layer, dropout layer, and regularisation term in the dense layers is to prevent overﬁtting to the limited training set [43]. In our proposed network, an enormous number of parameters may lead to overfining and failure during the network training. A dropout regularisation technique is introduced [46]. A subgroup of neurons with probability in a given layer will be eliminated as inactive neurons. These inactive neurons do not contribute to the feedforward and backpropagation processes. Under such conditions, because the active neurons cannot rely on the eliminated neurons, they are forced to learn more robust features independently. As a result, the network is well trained even with limited data. Batch normalization is a training technique for deep neural networks that normalizes each small batch of inputs to a single layer, with the effect of stabilizing the learning process and significantly reducing the number of training cycles needed to train a deep network.

#### 2.6.3. Adam Stochastic Optimization

The Momentum Optimization [47] was proposed to accelerate Stochastic Gradient Descent (SGD) [48]. This is achieved by reducing oscillations and directing SGD in the associated direction, at the cost of defining an additional hyperparameter. For this reason, Adam's algorithm, known as the adaptive moment [49], was used. An Adam optimization is relatively robust to the choice of hyperparameters during this implementation. A learning rate of 0.0001 was set, and Adam's default parameters were used to compute the first and second moments.

### 2.7. Evaluation Criteria

#### 2.7.1. Loss Function

Dice coefficient loss and cross-entropy loss are loss functions commonly used in semantic segmentation tasks. Former is an essential measure of the overlap between two samples. This measure ranges from 0 to 1, where a Dice coefficient of 1 means complete overlap, which is represented as equation (1), where *N* is the size of the pixels, *p*_*i*_ is the predicted pixels, and *y*_*i*_ is the test pixels. The cross-entropy loss examines each pixel one by one and compares the predicted results (probability distribution vector) for each pixel category with the heat-coded label vector. When there are only two categories, a binary entropy loss, called BCE loss, is used and represented as equation (2). However, the training is unstable when dealing with extremely unbalanced samples, both BCE and Dice coefficients. In BCE, if *y*=0 is much larger than *y*=1, then the *y*=0 component of the loss function will dominate, making the model heavily biased towards the background, resulting in poor training results. Therefore, the loss function in this study uses the combination of BCE and Dice loss; the formula is represented as equation (3), and the parameter *α* is used to control the weights of the BCE or Dice coefficients.

(1) $$\begin{array}{c} \text{Dice loss}=1-\frac{1}{N}\sum_{i=1}^{N}\frac{2\left|p_{i}\cap^{}y_{i}\right|}{\left|p_{i}\right|+\left|y_{i}\right|}, \end{array}$$

(2) $$\begin{array}{c} \text{BCE}=\frac{1}{N}\sum_{i=1}\left(y_{i}\log\;p_{i}\left(1-y_{i}\right)\log\left(1-p_{i}\right)\right), \end{array}$$

(3) $$\begin{array}{c} \text{loss}=\left(1-\alpha \right)\text{BCE}+\alpha \text{Dice}\times \text{loss,} \end{array}$$

(4) $$\begin{array}{c} \text{accuracy }=\;\frac{\text{TP}+\text{TN}}{\text{TP}+\text{TN}+\text{FP}+\text{FN}}. \end{array}$$

#### 2.7.2. Metrics

A confusion matrix was commonly used in the analysis of semantic segmentation. The confusion matrix was composed of (true positives (TP) false positives (FP), true negatives (TN), and false negatives (FN). The confusion matrix of TP, FP, FN, and TN is presented in Table 1. Methods were evaluated in terms of accuracy equation (4). All parameters range from 0 to 1 and are ideally as close to 1 as possible. In addition to the calculation of binary precision, the threshold parameter, which defaults to 0.5, is calculated. Each predicted value is compared with the threshold. Values greater than the threshold are set to 1, and values less than or equal to the threshold are set to 0.

The Dice coefficient is a commonly used indicator for evaluating segmentation result quality. It is mainly used to calculate the Dice distance of the two intervals to segment the similarity of the interval. The range is between 0 and 1. Dice loss is proposed to solve problems arising when the foreground proportion is too small. When the overlapping part of two samples is measured, the indicator ranges from 0 to 1 (where 1 represents complete overlap), which is defined as follows:

(5) $$\begin{array}{c} \text{Dice }=\;\frac{2\text{TP}}{\text{FN}+2\text{TP}+\text{FP}}. \end{array}$$

Intersection over Union (IoU) is a task outputting a prediction range. In order for IoU to be used to detect objects of any size and shape, it is necessary to mark the range of the detected object in the training set image and measure the correlation between the ground-truth and the prediction.

(6) $$\begin{array}{c} \text{IoU}=\;\frac{\text{TP}}{\text{TP}+\text{FP}+\text{FN}}. \end{array}$$

## 3. Experiment

### 3.1. Dataset

#### 3.1.1. International Skin Imaging Collaboration 2018 Dataset

The International Skin Imaging Collaboration (ISIC) has expert annotations on international datasets and is used to improve the automatic segmentation of melanoma diagnoses to help reduce mortality [50]. The ISIC-2018 dataset has a total of 5188 images, including skin lesion images and mask images. To evaluate our proposed method to larger datasets, we extract training data from the ISIC-2018: Skin Lesion Analysis Towards Melanoma Detection grand challenge dataset [50, 51]. Abraham and Mefraz Khan proposed a hybrid U-Net approach with a Dice score of 86% [52]. In this study, the proposed EfficientUNet++ has reached a Dice score of 96%; it displays superior performance to the previous reports. The detail results are described in the follow section.

#### 3.1.2. PH2 Dataset

PH2 is dataset of skin images obtained from the Pediatric Department of Pedro Hispano Hospital in Matosinhos, Portugal [53]. Data are provided on manual segmentation and clinical diagnosis. The dataset has a total of 400 images, including skin lesion images and mask images. In 2018, Yu proposed an aggregated deep convolutional features approach with a Dice score of 94% [6]. The proposed EfficientUNet++ has reached a Dice score of 96%, and it displays superior performance. The partitioning of datasets is essential for training models with generalization capabilities. The training and validation sets are used to train the model and to assess whether there is a good fit. The test set is used to test the model's performance with data that the model has unseen in model training phase.

### 3.2. Ablation Study

Ablation studies are used to analyze the performance of an artificial intelligence system by removing certain components to obtain an understanding of the contribution of that component to the overall system. The term is used by analogy with biology (the removal of components of an organism) and, continuing the analogy, especially when analysing artificial neural nets, by analogy with brain ablation procedures [54]. Meyes et al. [55] suggested that ablation studies are a viable approach to study knowledge representation in ANNs and are particularly useful to study the robustness of networks to structural damage, a feature of ANNs that will become increasingly important for future safety-critical applications [55]. To quantitatively verify the validity of our model, we performed ablation tests on PH2 data validation set (shown in Table 2). In our experiments, EfficientNet was used as the backbone [56], and submodules were added to perform the functional validation in the separate sessions.

The activation functions were also explored for ablation study. The chosen comparators are mainly the following activation functions: Mish, Swish, SELU, and ReLU [57]. For making the results more reliable, we only replaced the activation function of the network with Mish, Swish, GeLU, PReLU, and ReLU and kept the other hyperparameters unchanged. As shown in Table 3, the results revealed that the choice of ReLU as the activation function provided better accuracy than other functions.

### 3.3. Loss and Dice Coefficient Curves of Algorithms

**Figure 8:**
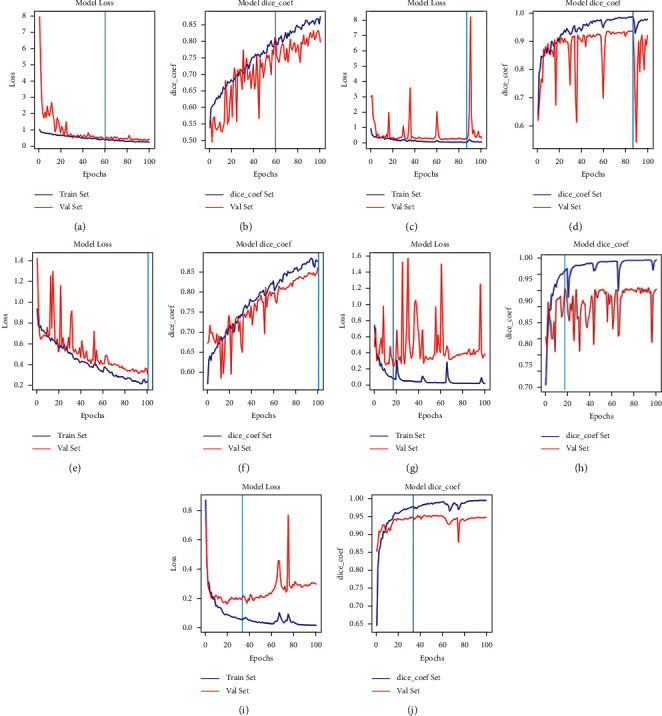
Validation set trends of loss and Dice coefficients for each method in the PH2 dataset. XceptionUNet and EfficientUNet++ appear the superior trends of loss and Dice coefficient than other models in the initial epochs. (a, b) SegNet, (c, d) U-Net, (e, f) UNet++, (g, h) XceptionUNet, and (i, j) EfficientUNet++.

**Figure 9:**
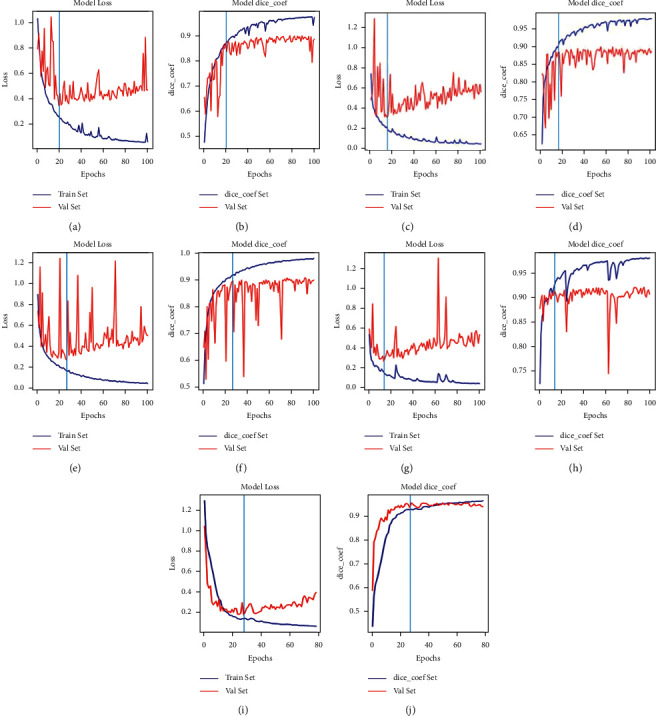
Validation set trends of loss and Dice coefficients for each method in the ISIC-2018 datasets. XceptionUNet and EfficientUNet++ appear the superior trends of loss and Dice coefficient to other models in the initial epochs. (a, b) SegNet, (c, d) U-Net, (e, f) UNet++, (g, h) XceptionUNet, and (i, j) EfficientUNet++.

As shown in Figures 8 and 9, our proposed method involves using 160 and 2570 training images of skin lesions from the PH2 and ISIC-2018 datasets, respectively. Early stopping (ES) is a common model training strategy in the literature. Jiménez and Racoceanu adopted ES to train the AlexNet and U-Net using stochastic gradient descent, a batch size of 128 and 100 epochs to mitosis analysis in breast cancer grading. They observe the convergence trend of the model, storing the weights before overfitting as the prediction model [58]. The training strategy is widely adopted in semantic segmentation and image recognition applications [59–61]. In this study, five models were trained, and then, tests were performed on 40 and 200 skin lesion images, respectively. For the loss of UNetEfficicent++ in Figures 8(i) and 8(j)), after 100 epochs of training, the steady increase in the loss function indicates that UNetEfficicent++ develops overfitting after 33th epoch. To overcome this issue, we used the weights saved in 33th epoch to test the performance of the network in the test dataset. For the loss of UNetEfficicent++ in Figures 9(i) and 9(j)), UNetEfficicent++was overfitted after the 27th epoch; as results, we used the weights saved in 27th epoch to test the performance of the network in the test dataset.

Five models were trained, and then, tests were performed on 40 and 200 skin lesion images, respectively, after 100 epochs of training. The ISIC-2018 and PH2 datasets also showed that the IoU and Dice scores improve with further increases in datasets and training steps. The ability of the proposed model to learn through experiments with the two datasets was evaluated using the accuracy curve shown in Figures 8 and 9. The curve demonstrates that the relatively large ISIC-2018 dataset reached a Dice coefficient percentage of 96%. This improvement is due to the Dice loss function used by the sigmoid classifier.

### 3.4. Evaluation of PH2 Dataset

**Figure 10:**
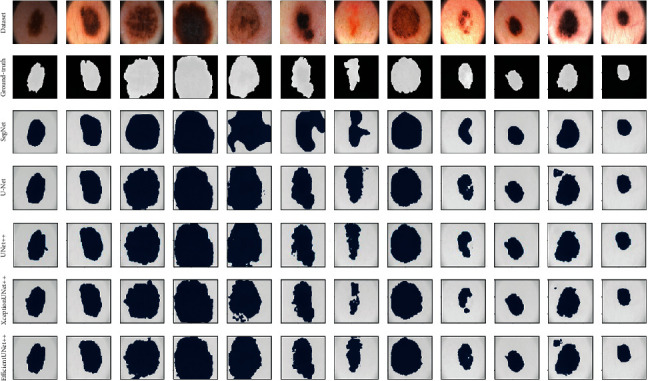
PH2 data-based model comparison of prediction results.

We compared the performance of our proposed method with that of other methods using the PH2 dataset. The results are listed in Table 4. Our method achieved the best performance. As shown in Figure 10 from the ground-truth and segmentation results, the SegNet algorithm performed relatively ineffective image segmentation on the PH2 dataset. Compared with UNet++, XceptionUNet++ increased by 2% and 3% with respect to Dice and IoU, and EfficientUNet++ increased by 4% and 4%, respectively. Compared with HRNeT, EfficientUNet++ increased by 2% with respect to Dice.

### 3.5. Evaluation of the ISIC-2018 Dataset

**Figure 11:**
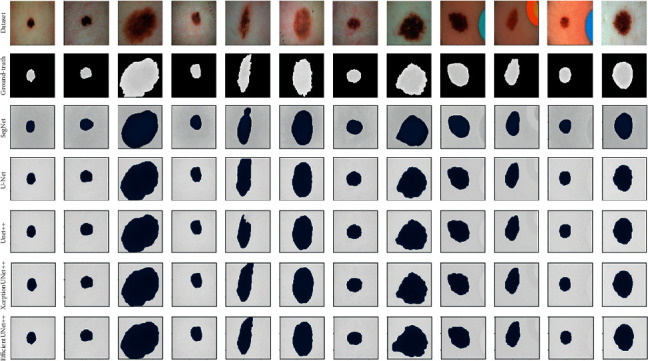
ISIC-2018 data-based model comparison of prediction results.

To verify the effectiveness of the proposed method, we compared the performance of various algorithms when applied to the ISIC-2018 dataset (Figure 11). The results are listed in Table 5. Our method achieved the highest scores in the default performance measures in this challenge compared with the other algorithms. The HRNeT and EfficientUNet++ have improvements of 2% and 3% when compared with UNet++ and XceptionUNet++. Even the novel HRNeT has a similar performance to EfficientUNet++, EfficientUNet++ shows a higher robustness and the best performance results on both datasets. In addition, EfficientUNet++ has fewer trainable parameters than HRNeT, which means that EfficientUNet++ is more efficient in memory usage than HRNeT (Table 6). As shown in Figure 11, the improved UNet++ algorithms made fuzzy boundaries clearer and enhanced focus on foreground pixels. Therefore, our method is capable of obtaining a high degree of precision and accuracy in pixel classification and segmentation, and it represents an improvement on other segmentation methods.

## 4. Discussion

Manual segmentation of skin lesion images is time consuming and imprecise. The deep learning image segmentation UNet++ algorithm can be used for the automatic segmentation of medical images; however, because of the density of connections, the number of calculations required is high, so UNet++ cannot accurately segment the locations and boundaries of skin lesions. Automatic lesion segmentation remains a challenge due to the large variation in the appearance of dermoscopic images, and streaks on dermoscopy images usually are difficult to detect because they are not perfect linear structures b[[parms resize(1),pos(50,50),size(200,200),bgcol(156)]] lighting condition, and were subject to nonuniform vignetting [62]. Sample imbalance in the PH2 dataset [63–65] causes segmentation models to be severely biased and results in low prediction accuracy. Although this problem can be overcome through data enhancement, the possible improvement is limited. The most direct solution is to expand the size of the original dataset or use a focal loss function [66] suitable for the unbalanced sample. However, focal loss has static loss that does not change with data distribution, it failed to meet expectations due to instability during training. Therefore, we use the loss function that adopts the combination of Dice loss and BCE loss, realizing the accurate segmentation of skin lesion.

The preprocessing of input images is very helpful in the task of segmentation, which consists of working with grayscale images and normalizing to improve image quality. In the UNet++ model, quality of encoding affects the final segmentation. Due to time and calculation limitations, it is unfeasible to train bespoke models from scratch. In this study, we proposed an improved UNet++ algorithm in which Xception and EfficientNet pretraining is implemented. Using pretrained image networks on the encoder can be useful because pretraining reduces the neural network model training time and can reduce errors. Pretrained models have learned to extract powerful and useful functions from images and use them as starting points for learning new tasks, and they can use pretraining as a benchmark from which to improve existing models. However, UNet++'s dense connection and the memory usage are efficient; meanwhile, the network can avoid vanishing gradient. However, this approach causes excessive irrelevant features passing on the network and the redundant use of computational resources. Therefore, to mitigate feature map explosion in the upsampling path, the dense connections of UNet++ are avoided in the network structure and improved the way to skip connection. The improved UNet++ algorithm can improve accuracy and efficiency in medical image segmentation. We used five deep learning algorithms for comparative experiments and performed image segmentation on the PH2 and ISIC-2018 datasets to verify the effectiveness of the improved method. The results show that the segmentation of the improved UNet++ is superior to that of other algorithms.

For modelling the features of melanoma skin lesion, EfficientUNet++ can exploit the advantages of EfficientNet and UNet++, and the performance of the method proposed in this study is verified in both ablation study and 5-fold cross validation. The primary contributions are concluded as follows:

1. An effective semantic segmentation model based on deep learning is proposed and validated with two skin lesion data
2. The validity of the proposed method is compared with that of four classical models: SegNet [32], U-Net [31], UNet++ [32, 33], Xception [37], and the state-of-the-art method HRNeT [27]
3. The proposed method exhibits the strengths from both of EfficientNet and UNet++ features
4. The experiments demonstrated that the robustness of EfficientUNet++ has outperformed other methods
5. This work verified that the practicability of the integrated method of UNet ++ and EfficientNet

Although our model has achieved promising segmentation accuracy in two independent datasets, the lighter colors in lesion areas were not accurately segmented; therefore, our model requires further improvements. In the future, we will attempt to join residual blocks [67] and SE blocks [68], and these challenging topics deserve further study.

## 5. Conclusions

Manual image segmentation may result in errors and inefficiency. Therefore, an automatic image segmentation algorithm can help doctors to diagnose the size and location of melanoma lesions and reduce medical costs. To improve the UNet++ algorithm, we propose EfficientUNet++. This method combines UNet++ and EfficientNet networks and redesigns skip connections to aggregate features of varying semantic scales at the decoder subnetworks, leading to a highly flexible feature fusion scheme, thereby accelerating network convergence and retaining more edge information. The EfficientUNet++ algorithm structurally expands the size of the convolutional network by simply and efficiently compositing coefficients, and the introduction of residual units deepens the network and thereby improves performance. As a result, the outputs of EfficientUNet++'s PH2 and ISIC-2018 datasets are more accurate than those of other methods. Verification can prove that skin lesions can be well segmented using the improved UNet++. This research can play a vital role in reducing manual interventions and misdiagnoses, improving accuracy and solving related medical image segmentation problems. However, due to the dataset's limitation, the model's capacity to identify melanoma lesions was restricted, and the proposed method does not provide further insight into the prognosis of melanoma lesions that have received attention in recent studies [69, 70]. Recently, the novel techniques for jointing multisegmentation of multiscale feature extraction have been proposed [71, 72]. In the future studies, the new technique can be combined to enhance the model's capacity to aware boundary for melanoma lesions and add temporal image data to investigate the model for the prediction of melanoma lesions' prognosis.

## Figures and Tables

**Table 1:** Confusion matrix for binary classification.

Prediction result	Actual test	
	Positive	Negative
Positive	TP	FP
Negative	FN	TN

**Table 2:** Ablation study results for the different modules in PH2 data.

Method	Dice	Accuracy	IoU
EfficientNet + U-Net	0.90	0.92	0.90
EfficientNet + inception	0.90	0.93	0.91
EfficientNet + xception	0.91	0.93	0.92
EfficientNet + UNet++	**0.93**	**0.96**	**0.96**

Note: the best results are in bold.

**Table 3:** Results of the ablation study with different activation functions.

Activation function	Epoch	Learning rate	Batch size	Optimizer	Backbone	Accuracy
Mish	100	0.001	2	Adam	EfficientNet	0.92
Swish	100	0.001[[parms resize(1),pos(50,50),size(200,200),bgcol(156)]]	2	Adam	EfficientNet	0.91
GeLU	100	0.001	2	Adam	EfficientNet	0.92
PReLU	100	0.001	2	Adam	EfficientNet	0.94
ReLU	100	0.001	2	Adam	EfficientNet	**0.96**

Note: the best results are in bold.

**Table 4:** Model performance regarding PH2 data.

Method	Dice	Accuracy	IoU
HRNeT	0.91	**0.96**	**0.96**
SegNet	0.76	0.94	0.74
U-Net	0.89	0.94	0.82
UNet++	0.89	**0.96**	0.92
XceptionUNet++	0.91	**0.96**	0.95
EfficientUNet++	**0.93**	**0.96**	**0.96**

Note: bold indicates the best results.

**Table 5:** Model performance regarding ISIC-2018 data.

Method	Dice	Accuracy	IoU
HRNeT	**0.96**	0.97	**0.94**
SegNet	0.94	0.95	0.89
U-Net	0.95	0.95	0.92
UNet++	0.95	0.96	0.91
XceptionUNet++	**0.96**	**0.98**	0.93
EfficientUNet++	**0.96**	**0.98**	**0.94**

Note: bold indicates the best results.

**Table 6:** Trainable parameters regarding models.

Method	Trainable parameter
HRNeT	9,504,578
SegNet	33,393,157
U-Net	487,289
UNet++	5,223,107
XceptionUNet++	38,370,009
EfficientUNet++	6,653,549

## Data Availability

The data used to support the findings of this study are available from the corresponding author upon request.
